# Clinicopathological characteristics and outcomes in men with mesothelioma of the tunica vaginalis testis: analysis of published case-series data

**DOI:** 10.1007/s00432-021-03533-6

**Published:** 2021-02-09

**Authors:** Josias Bastian Grogg, Jordi Nicola Fronzaroli, Pedro Oliveira, Peter-Karl Bode, Anja Lorch, Allaudin Issa, Joerg Beyer, Daniel Eberli, Vijay Sangar, Thomas Hermanns, Noel William Clarke, Christian Daniel Fankhauser

**Affiliations:** 1 Department of Urology, University Hospital Zurich, Frauenklinikstrasse 10, 8091 Zurich, Switzerland; 2 The Christie NHS Foundation Trust, Manchester, UK; 3 Inselspital Bern, University of Bern, Bern, Switzerland; 4 The Salford Royal NHS Foundation Trust, Manchester, UK

**Keywords:** Urology, Testis cancer, Mesothelioma, Orchiectomy, Testis-sparing surgery, Systematic review

## Abstract

**Purpose:**

Mesothelioma of the tunica vaginalis testis (MTVT) is a rare tumor, and currently, there are no published treatment recommendations.

**Methods:**

We performed a systematic literature review and synthesized clinical presentation, clinicopathological factors associated with metastatic disease, treatment options, and outcomes in men with MTVT.

**Results:**

We included 170 publications providing data on 275 patients. Metastatic disease occurred in 84/275 (31%) men with malignant MTVT: Most common sites included retroperitoneal lymph nodes (LNs) (40/84, 48%), lungs (30/84, 36%), and inguinal LNs (23/84, 27%).

Invasion of the spermatic cord or scrotum was the only risk factor for local recurrence [odds ratio (OR) 3.21, 95% confidence interval (CI) 1.36–7.57]. Metastatic disease was associated with age ≥ 42 years (OR 3.02, 95% CI 1.33–6.86), tumor size ≥ 49 mm (OR 6.17, 95% CI 1.84–20.74), presence of necrosis (OR 8.31, 95% CI 1.58–43.62), high mitotic index (OR 13.36, 95% CI 1.53–116.51) or angiolymphatic invasion (OR 3.75, 95% CI 1.02–13.80), and local recurrence (OR 4.35, 95% CI 2.00–9.44). Complete remission in the metastatic setting was observed in five patients, most of whom were treated with multimodal therapy. Median survival in patients with metastatic disease was 18 months (IQR 7–43).

**Conclusion:**

Malignant MTVT is a rare but aggressive disease. Since local recurrence is a risk factor for metastatic progression, we recommend aggressive local treatment. Survival and response to any treatment in the metastatic setting are limited.

**Supplementary Information:**

The online version contains supplementary material available at 10.1007/s00432-021-03533-6.

## Introduction

Mesothelioma typically involves the pleura or peritoneum and only rarely the tunica vaginalis testis (Gurdal and Erol 2001; Plas et al. 1998a). Due to the low prevalence of mesothelioma of the tunica vaginalis testis (MTVT), treatment recommendations have not been developed. We collated published case reports of men with MTVT to describe clinicopathological factors associated with local recurrence or metastatic disease as well as outcome from treatments used.

## Methods

### Evidence acquisition

#### Data acquisition and search strategy

A systematic literature review was performed using the Preferred Reporting Items for Systematic Reviews and Meta-analysis (PRISMA) statement (Moher et al. 2009, 2015). Prior to data acquisition, the study protocol/search strategy was published on the PROSPERO registry (http://www.crd.york.ac.uk/PROSPERO; Registration Number CRD42019129595).

Our search identified articles published up to 14th January 2019 and utilised the following electronic databases: MEDLINE^®^, Embase^®^, Scopus^®^, the Cochrane Database of Systematic Reviews, and Web of Science. To identify all relevant articles, a clinical medical librarian applied a broad approach using several combinations, synonyms and related search terms to “para-/intra-/testicular mesothelioma”, “epididymal mesothelioma”, or “para-/intra-/testicular mesenchymal tumor/neoplasm”. Non-English literature was excluded unless the abstract was available in English or the full text in French, Spanish, Italian, Portuguese, or German. Moreover, the reference lists of the identified publications were screened manually to identify additional studies. For the search strategy, see Supplementary File 1. After deduplication, two authors (JBG, JNF) screened the titles and abstracts independently and data from the same study that appeared in multiple publications were included only once. Finally, disagreements were discussed and resolved by consensus or by third-party arbitration (CDF).

#### Types of outcome measures included and data extraction

Studies containing data on patient characteristics, clinicopathological features, treatment of local or metastatic disease, site of metastases, and follow-up information were eligible for this review. The data extraction sheet was developed according to the Cochrane Consumers and Communication Review Group’s data extraction template. Whenever possible, data were gathered at the single-patient-level.

#### Statistical analysis

Categorical variables are presented as percentages and were compared using the Chi-square test of independence. The assessment of normality for continuous variables was performed visually and using the Shapiro–Wilk test. The results for normally distributed variables are expressed as mean ± standard deviation (SD), while non-normally distributed variables are presented as median and interquartile ranges (IQRs). The independent samples *t* test was used for normally distributed data and the Mann–Whitney *U* test for variables with non-normally distributed data. Weighted medians were used to estimate previously processed statistical data from cohort studies as well as individual patient data from single-case reports. The optimal cut-off value for continuous variables was determined using receiver-operating curve (ROC) analyses and the maximal Youden's index (= Sensitivity + Specificity– 1) (Youden 1950). All *p* values < 0.05 were considered statistically significant; all statistical tests were two-sided.

## Results

### Included studies

**Fig. 1:**
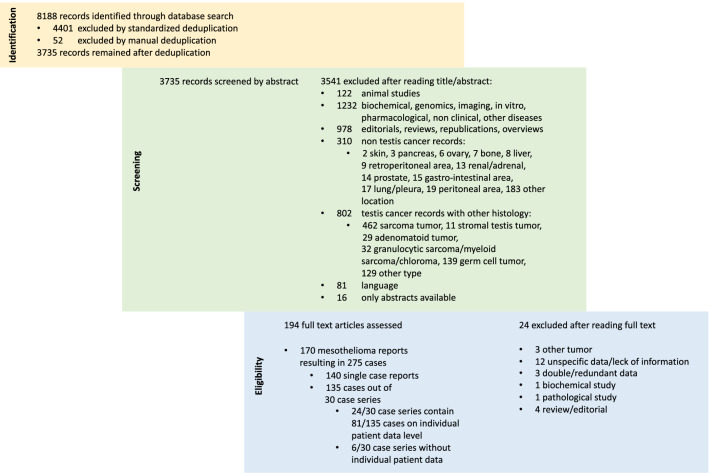
Flowchart of the study selection process

After deduplication and title as well as abstract screening, 194 of the originally identified 8,188 publications were eligible for full-text review (Fig. 1). We finally included 170 studies, resulting in a dataset of 275 patients, consisting of 140 single-case reports and 30 case series.

### Subgroups and location, demographics, clinical symptoms, and laboratory findings

We identified 275 cases of MTVT, of which 227/275 (83%) were classified as “malignant mesothelioma”, with a histopathological sub-classification including epithelioid (130/227, 57%), sarcomatoid (4/227, 2%), and biphasic subtype (53/227, 23%) and malignant mesothelioma of an unknown subtype (40/227, 18%). Other histologies included well-differentiated papillary mesothelioma (30/275, 11%) and mesothelioma of uncertain malignant potential (13/275, 5%). Cases of benign cystic mesotheliomas (5/275, 2%), also known as multilocular inclusion cysts, which typically develop in the peritoneum, were also included (Table 1). The median age at diagnosis was 62 years (IQR 44–73). The tumor invaded the tunica vaginalis as the only affected structure in 170/275 patients (62%); for the remaining patients, the tunica vaginalis plus other (multiple) structures were involved: tunica albuginea and/or testis in 55/275 (20%); epididymis in 34/275 (12%), spermatic cord in 65/275 (24%), and the scrotum (tunica dartos and dermis) in 20/275 (7%).

**Table 1:** Patient characteristics overall and on individual patient-level data

	Individual patient level of 164 reports*	Study-level data from 170 reports
Number of patients	221 (= 100%)	275 (= 100%)
Histopathological (sub-)types		
Malignant MTVT (%)	180/221 (81%)	227/275 (83%)
Epithelioid type (% of malign. MTVT)	100/180 (56%)	130/227 (57%)
Sarcomatoid type (% of malign. MTVT)	4/180 (2%)	4/227 (2%)
Biphasic type (% of malign. MTVT)	40/180 (22%)	53/227 (23%)
Malignant MTVT of unknown subtype (% of malign. MTVT)	36/180 (20%)	40/227 (18%)
Mesothelioma of uncertain malig. potential (%)	13/221 (6%)	13/275 (5%)
Well differentiated papillary MTVT (%)	23/221 (10%)	30/275 (11%)
Benign cystic mesothelioma (%)	5/221 (2%)	5/275 (2%)
Missing (%)	0/221 (0%)	0/221 (0%)
Age (years)		
Median (IQR)	62 (44–73)	–
Missing (%)	1/221 (< 1%)	
Side		
Left (%)	105/221 (48%)	127/275 (46%)
Right (%)	98/221 (44%)	107/275 (39%)
Bilateral (%)	5/221 (2%)	5/275 (2%)
Missing (%)	13/221 (6%)	36/275 (13%)
Clinical presentation	209/221 (95%)	259/275 (94%)
Testicular enlargement/swelling/mass (%)	200/209 (96%)	244/259 (94%)
Scrotal pain/discomfort (%)	40/209 (19%)	40/259 (15%)
Epididymitis/orchitis (%)	13/209 (6%)	13/259 (5%)
Lymphadenopathy (%)	7/209 (3%)	7/259 (3%)
Incidental finding during scrotal surgery (%)	87/209 (42%)	93/259 (36%)
Missing (%)	12/221 (5%)	16/275 (6%)
Exposition		
Asbestos (%)	146/221 (66%)	154/275 (56%)
Yes (%)	51/146 (35%)	59/154 (38%)
No (%)	95/146 (65%)	95/154 (62%)
Smoking (%)	11/221 (5%)	11/275 (4%)
Mean pack years (SD)	21 (± 13)	–
Median pack years (IQR)	23 (12–29)	–
Missing (%)	74/221 (33%)	121/275 (44%)
Tumor markers		
AFP elevated (%)	1/52 (2%)	1/52 (2%)
HCG elevated (%)	2/52 (4%)	2/52 (4%)
LDH elevated (%)	3/52 (6%)	3/52 (6%)
Missing (%)	168/221 (76%)	168/275 (61%)
Local primary treatment		
Radical inguinal orchiectomy (%)	136/221 (62%)	170/275 (62%)
Testis-sparing surgery (%)	41/221 (19%)	52/275 (19%)
Radical hemiscrotectomy (%)	26/221 (12%)	28/275 (10%)
Hydrocelectomy only (%)	8/221 (4%)	8/275 (3%)
Fine needle aspiration only (%)	3/221 (1%)	3/275 (1%)
Transscrotal orchiectomy (%)	2/221 (< 1%)	2/275 (< 1%)
Missing (%)	5/221 (2%)	12/275 (4%)
Local secondary treatment		
Orchiectomy after TSS (% of TSS)	13/41 (32%)	13/52 (25%)
Hemiscrotectomy after TSS (% of TSS)	6/41 (15%)	6/52 (12%)
Hemiscrotectomy after Orchiectomy (% of RIO)	3/41 (7%)	3/52 (6%)
Histopathology features		
Necrosis (%)	27/221 (12%)	31/275 (11%)
Angiolymphatic invasion (%)	33/221 (15%)	40/275 (15%)
Mitotic activity (%)	53/221 (24%)	59/275 (21%)
High mitotic activity (> 3/10 HPF) (%)	26/221 (12%)	32/275 (12%)
Missing (%)	143/221 (65%)	174/275 (63%)
Size (mm)		
Median of all histologies (IQR)	35 (15–70)	–
Malignant MTVT (IQR)	40 (15–80)	
Epithelioid type (IQR)	38 (14–100)	
Biphasic type (IQR)	55 (27–100)	
Well-differentiated papillary MTVT (IQR)	15 (10–29)	
Radical inguinal orchiectomy (IQR)	38 (13–78)	–
Testis-sparing surgery (IQR)	24 (12–63)	–
Radical hemiscrotectomy (IQR)	40 (30–150)	–
Missing (%)	148/221 (67%)	

*MTVT* mesothelioma of the Tunica Vaginalis Testis, *RIO* radical inguinal orchiectomy, *TSS* testis-sparing surgery, *AFP* alpha-fetoprotein, *HCG* human chorionic gonadotropin, *LDH* lactate dehydrogenase, *IQR* inter quartile range

*This column includes only patients of which individual patient-level data was available

### Clinical presentation

Data regarding clinical presentation were available for 259/275 patients (94%) (Table 1). Most patients presented with a painless testicular mass/swelling, or a hydrocele (244/259, 94%). Scrotal pain was described in 40/259 cases (15%), and inflammation with orchitis or epididymitis in 13/259 (5%). Incidental findings during scrotal surgery represented 93/275 cases (36%). We found no reports suggesting hormonal changes leading to gynecomastia or earlier/later puberty. Of the 154 patients with information about potential environmental risk factors of mesothelioma, 59 (38%) described exposure to asbestos, while smoking was mentioned by 11 patients (4%). Of the 52 patients with specific information on testicular tumor markers, lactate dehydrogenase (LDH) was elevated in 3/52 cases (6%), human chorionic gonadotropin (HCG) in 2/52 cases (4%), and alpha-fetoprotein (AFP) in 1/52 cases (2%).

### Local treatment and recurrence

Most patients (170/275, 62%) underwent radical inguinal orchiectomy as the primary local treatment, while testis-sparing surgery (TSS) was performed in 52/275 cases (19%). Primary hemiscrotectomy was reported in 28/275 (10%), transscrotal orchiectomy in two (< 1%), hydrocelectomy as the only surgical intervention in eight (3%), and fine needle aspiration (FNA) as the diagnostic procedure without any further treatment in three (1%). After primary TSS, secondary completion orchiectomy and secondary completion hemiscrotectomy were performed in 13 (25%) and 6 patients (12%), respectively. After primary orchiectomy, secondary hemiscrotectomy was performed in three (6%) patients. In 12 (4%), the primary therapy was not reported. Of the 203 patients with available follow-up data, 117 (58%) displayed no evidence of disease after a median of 19 (IQR 9–41) months. By contrast, 67 (33%) died after a median of 20 months (IQR 7–48). 19 men (9%) were alive with disease after a median of 21 months (IQR 8–24). Local recurrence was observed in 35/275 patients (13%) (Supplementary Table 1). Of the 32 patients for whom follow-up time was available, the median time from diagnosis to local recurrence was 17 months (IQR 4–24 months). The following recurrence rates were reported for the different primary treatment options: radical inguinal orchiectomy: 24/170 (14%) after a median of 10 months (IQR 3–24), TSS: 6/52 (12%) after a median of 24 months (IQR 7–30), and radical hemiscrotectomy: 3/28 (11%) after a median of 24 months (IQR 19–55). Local recurrence in benign multicystic mesothelioma or MTVT of uncertain malignant potential was observed in one case after TSS and transscrotal orchiectomy, respectively. Salvage treatment was successful in seven patients and included local resection of recurrence and orchiectomy in five and two men with MTVT, respectively. In univariable regression analyses, only invasion of the spermatic cord or scrotum was associated with local recurrence (OR 3.21, 95% CI 1.36–7.57, *p* = 0.008) (Table 2).

**Table 2:** Univariable regression analyses for local recurrence and metastatic disease

Variable		Local recurrence			Metastatic disease		
	# With available variable (% of 221*)	OR	CI	*p* value	OR	CI	*p*-value
Age (≥ 42 years)	211 (95)	2.47	0.82–7.42	0.107	3.02	1.33–6.86	0.009
Tumor size (≥ 49 mm)	67 (30)	3	0.76–11.92	0.119	6.17	1.84–20.74	0.003
Histology Malignant MTVT Epithelioid type Sarcomatoid type Biphasic type MTVT of uncertain malignant potential	164 (74) 151 (68) 97 4 38 13 (8)	Reference 0.00 0.82 0.37	– – 0.30–2.26 0.05–3.00	0.814 0.999 0.706 0.349	Reference 0.00 1.59 0.15	– – 0.75–3.38 0.02–1.24	0.167 0.999 0.230 0.079
Necrosis	45 (20)	1.13	0.22–5.79	0.880	8.31	1.58–43.62	0.012
High mitotic index	43 (19)	6.26	0.70–56.29	0.101	13.36	1.53–116.51	0.019
Angiolymphatic invasion	50 (23)	1.26	0.20–7.64	0.802	3.75	1.02–13.80	0.047
Location/involvement Tunica vaginalis only Invasion of tunica albuginea and/or testis Invasion of epididymis Invasion of spermatic cord or scrotum	211 (95) 121 (55) 23 (10) 19 (9) 48 (22)	Reference 2.47 0.99 3.21	– 0.78–7.80 0.20–4.76 1.36–7.57	0.044 0.124 0.986 0.008	Reference 0.61 0.78 1.56	– 0.21–1.76 0.26–2.32 0.78–3.11	0.335 0.357 0.655 0.207
Local recurrence							
Recurrence versus no recurrence	209 (95)	–	–	–	4.35	2.00–9.44	< 0.001

*OR* odds ratio, *CI* confidence interval, *MTVT* mesothelioma of the tunica vaginalis testis, *TSS* testis-sparing surgery

*Cases with individual patient-level data

### Adjuvant therapy

Lymph-node dissection (LND) was performed in 21 men of whom 12 had inguinal LND, 8 retroperitoneal LND, 7 pelvic LND, and 6 LND in multiple locations. In 5 of the 21 patients (24%), histological analysis showed lymph-node metastases (LNs), one without recurrence during follow-up. Two patients were alive with disease (one with ongoing chemotherapy and one with no further therapeutic interventions). Two patients died from metastatic spread after palliative radiotherapy and radiochemotherapy, respectively. An additional three patients without positive lymphadenectomy results displayed evidence of distant recurrence during follow-up. Thus, 6 of the 21 patients (29%) developed systemic disease despite adjuvant LND. Local and/or metastatic relapse after adjuvant chemotherapy or chemoradiotherapy was observed in 5/9 (56%) patients. Used chemotherapy agents included Doxorubicin, Carboplatin, or combination therapy with Adriamycin plus Cyclophosphamide or Paclitaxel, and the radiation field included the retroperitoneal and pelvic lymph nodes and pelvic region. 6/11 (55%) men relapsed after several radiotherapy protocols including radiation of the scrotum as well as the retroperitoneal and inguinal LNs with maximum doses between 55 and 60.5 Gy.

### Onset and site of metastasis

**Fig. 2:**
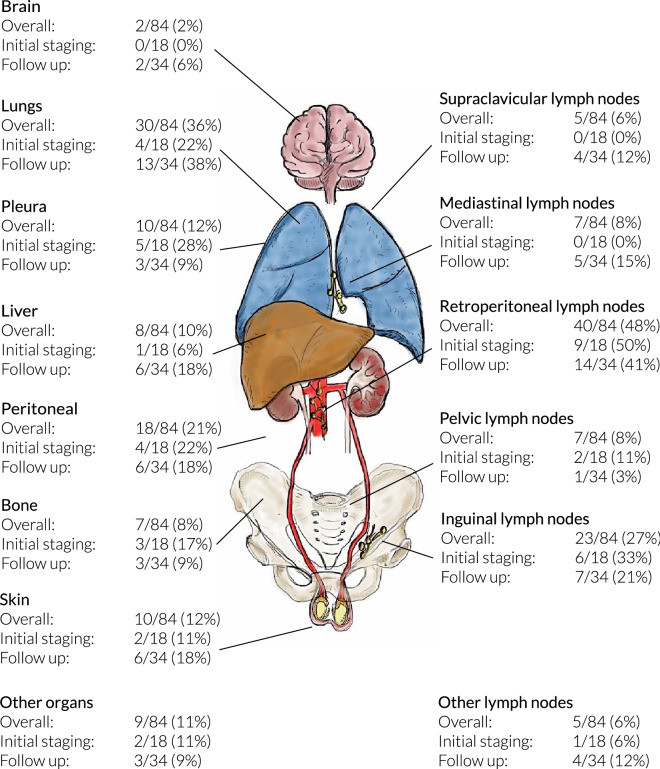
Anatomical locations of metastatic sites at initial staging and during follow-up. Affected organs are listed on the left and lymph-node locations on the right

Overall, metastatic disease was observed in 84/275 patients (31%). The following sites of metastasis were described: Retroperitoneal lymph nodes (RPLNs) (40/84, 48%); lungs (30/84, 36%); inguinal LNs (23/84, 27%); peritoneal surfaces (18/84, 21%); pleura (10/84, 12%); skin (10/84, 12%); liver (8/84, 10%); pelvic LNs, mediastinal LNs and bones (each 7/84, 8%); supraclavicular LNs (5/84, 6%); abdominal LNs (4/84, 5%); brain, kidneys, bladder, or pancreas (each 2/84, 2%), colon, rectum, spleen, and cervical LNs (each 1/84, 1%) (Fig. 2). Regarding the subgroup of 29/84 patients (35%) with only one metastatic site, the RPLNs (7/29, 24%) and lungs (7/29, 24%) were the most common primary sites. Other solitary metastatic sites included the peritoneum (4/29, 14%), inguinal LNs, and bones (3/29, 10% each). Two case reports each (2/29, 7%) described singular metastasis of MTVTs to the brain, the liver, and the mediastinal LNs. The exact timing of metastatic disease in relation to diagnosis was described in 52/84 (62%) patients. Metastatic disease at initial diagnosis was described in 18/52 men (35%), whereas 34/52 men (65%) presented with metastatic recurrence during a median follow-up of 17 months (IQR 10–29).

### Risk factors for metastatic disease

Patients with well-differentiated papillary mesothelioma or benign multicystic mesothelioma did not develop metastatic disease. Regarding the different histologic sub-classifications of malignant MTVT on individual patient-level data, 34/100 patients (34%) with epithelioid type and 18/40 (45%) with biphasic type exhibited metastatic behaviour. The remaining patients with metastatic disease were either unclassified or belonged to other rare subtypes of malignant MTVT [15/36 (42%) malignant MTVT without subtype information, 1/13 (8%) MTVT of uncertain malignant potential].

Individual patient-level data to analyse further risk factors were available for 68 men with metastatic MTVT. Risk factors for metastasis included age ≥ 42 years (OR 3.02, 95% CI 1.33–6.86, *p* = 0.009), tumor size ≥ 49 mm (OR 6.17, 95% CI 1.84–20.74, *p* = 0.003), and presence of necrosis (OR 8.31, 95% CI 1.58–43.62, *p* = 0.012), high mitotic index (OR 13.36, 95% CI 1.53–116.51, *p* = 0.019), or angiolymphatic invasion (OR 3.75, 95% CI 1.02–13.80, *p* = 0.047), and local recurrence (OR 4.35, 95% CI 2.00–9.44, *p* < 0.001).

### Treatment and outcome in men with metastatic disease

During a median follow-up of 18 months (IQR 7–43), 55/84 metastatic MTVT patients (65%) died, and 13/84 (15%) were alive with disease, while 11 patients were either lost to follow-up or without information on survival. Complete remission in the metastatic setting was reported in five men (Supplementary Table 2): the first patient (#7) with an epithelial MTVT was treated with an orchiectomy and RPLND showing involvement of one retroperitoneal lymph node. Data about staging CT at diagnosis as well as information about further treatment during follow-up were not available. This patient remained free of disease for at least 66 months (Bertolotto et al. 2016). The second patient (#12) with an epithelioid MTVT developed mediastinal LN metastases 36 months after primary radical orchidectomy. After six cycles of Cisplatin and Pemetrexed and maintenance with Pemetrexed, there was no evidence of disease after 42 months of follow-up (Doris et al. 2015). The third patient (#35), with a malignant MTVT of unknown histological subtype and peritoneal metastases at initial staging, was treated with testis-sparing surgery, peritonectomy, and intraperitoneal hyperthermic perfusion chemotherapy. He remained free of disease after a follow-up of 18 months (Schure et al. 2006). The fourth patient (#42), who had epithelioid MTVT with extension to the spermatic cord in the inguinal canal and metastatic spread within the pelvis and the abdomen, was initially treated with hemiscrotectomy with resection of the inguinal mass and abdominal cytoreductive surgery with extensive stripping all peritoneal surfaces followed by intra-operative, intra-peritoneal chemotherapy using Cisplatin and Doxorubicin. This patient was recurrence-free survival at 5 years (Sebbag et al. 2001). The fifth patient (#60), with epithelioid MTVT invading the tunica albuginea, the spermatic cord and external scrotal layers, was treated with a primary hemiscrotectomy. After resection of visible metastases in the retroperitoneum, six cycles of chemotherapy with Adriamycin and Cyclophosphamide and radiation therapy of 25 Gy over 3 weeks, the patient was disease-free at 36 months (Lopez et al. 1995). Of metastatic cases with available follow-up, 20 out of 21 (95%) patients treated with surgery only and 64 out of 68 (94%) patients treated with multimodal therapies progressed.

## Discussion

Our analysis of published case series is the largest and most comprehensive summary of the available literature regarding MTVT. It also extends the previous reviews (Bisceglia et al. 2010; Plas et al. 1998b; Vimercati et al. 2019; Zhang and Goldsztajn 2019), and therefore, several important insights can be discussed.

The highest incidence was found in men in their sixties; however, there is a wide age range, even patients under 20 years can be affected. MTVT usually presents as a testicular mass or is incidentally discovered during or after inguino-scrotal surgery. Therefore, the primary local treatment depends on the clinical scenario, but treatment should also be planned in the knowledge that MTVT can show multifocal growth and that local recurrence might be associated with metastatic disease.

In men with first presentation, several recommendations might help to guide clinicians in decision-making, but those are based on very limited evidence or expert opinion only. Men in whom MTVT is suspected at initial presentation, primary hemiscrotectomy, and en-bloc orchidectomy should be performed. In men with suspicious findings discovered during surgery, we recommend completing the hydrocelectomy as planned and only proceeding thereafter to a hemiscrotectomy if the final histopathology confirms the diagnosis of MTVT. This is mainly since most men scheduled for routine scrotal surgery such as hydrocelectomy are not consented for a hemiscrotectomy and the diagnostic accuracy of intra-operative frozen section remains ill-defined. For men with an incidental diagnosis of MTVT in the hydrocelectomy specimen, we recommend timely completion of a hemiscrotectomy because of potential tumor seeding.

Well-differentiated papillary mesothelioma and benign multicystic mesothelioma are clearly benign entities that require complete resection as definitive therapy; thereafter, only follow-up for local relapse is advisable. By contrast, malignant MTVT exhibited metastatic spread in one-third of cases, suggesting repeated cross-sectional imaging of the chest and abdomen is required for staging and follow-up. The available data do not support the regular use of adjuvant treatment. Half of all cases had involved retroperitoneal LNDs, and only in a proportion, other organs were also affected. Despite the use of RPLND, all patients treated with lymphadenectomy, who did not receive additional chemo- or radiotherapy, progressed. Thus, RPLND as monotherapy does not have the potential to cure patients. Only a few men received adjuvant chemo- or radiotherapy which does not allow any conclusions.

Patients with metastatic disease have a poor prognosis, and standard treatment recommendations are not available. Given the experience in pleural mesothelioma and the scarce data on MTVT, surgical resection within a multimodal treatment approach can be offered; however, the choice of chemotherapy and the role of radiotherapy remain unclear. Pleural mesothelioma guidelines recommend Platinum, Pemetrexed plus minus Bevacizumab (Opitz et al. 2020) and in one case Pemetrexed led to complete response. Additionally, Cisplatin and Doxorubicin or Adriamycin and Cyclophosphamide could be suggested as a few responses have been observed.

### Limitations

The published literature only consists of retrospective case reports and small case series with a low number of outcome events and missing single-patient data, and multivariable analysis was therefore not possible. Our search strategy was designed and reviewed by both clinicians and librarians, and was predefined in a peer-reviewed protocol. However, the possibility remains that not all potentially relevant studies were identified; this is an additional source of potential bias. Larger and more consistent datasets are needed to develop prediction models involving several risk factors. Nevertheless, the current analysis provides a unique overview of the published experience with MTVTs. Due to the absence of prospective trials, we recently opened the OrphAn Testis Histologies (OATH) to provide more conclusive recommendations regarding clinical course, management, and follow-up of these rare entities. We encourage collaborators to contribute data regarding patients with rare testis cancer histologies (http://bit.ly/OATH-registry).

## Supplementary Information

Below is the link to the electronic supplementary material.

Supplementary file1 (DOCX 32 KB)

Supplementary file2 (DOCX 46 KB)

Supplementary file3 (PDF 60 KB)

## Data Availability

The datasets used and/or analysed during the current study are available from the corresponding author on reasonable request.

## Abbreviations

MTVT: Mesothelioma of the tunica vaginalis testis
TSS: Testis-sparing surgery
PRISMA: Preferred Reporting Items for Systematic Reviews and Meta-analysis
IQR: Interquartile range
SD: Standard deviation
CI: Confidence interval
ROC: Receiver-operating curve
AFP: Alpha-1 fetoprotein
HCG: Human chorionic gonadotropin
LDH: Lactate dehydrogenase
RPLN: Retroperitoneal lymph nodes
ILN: Inguinal lymph nodes
PLN: Pelvic lymph nodes
LND: Lymph-node dissection
HPF: High-power field
BEP: Bleomycin, etoposide, and cisplatin
DC: Doxorubicin–cisplatin
OATH: OrphAn testis histologies
