# Supplementary material for: Clinicopathological characteristics and outcomes in men with mesothelioma of the tunica vaginalis testis: analysis of published case-series data
Source: J Cancer Res Clin Oncol. 2021 Feb 9;147(9):2671–9. doi: 10.1007/s00432-021-03533-6 (PMC8310841; doi:10.1007/s00432-021-03533-6)
Supplement: Supplementary file 1 — Supplementary file1 (DOCX 32 KB) [file 432_2021_3533_MOESM1_ESM.docx]

**Supplementary table 1: Features and treatment outcomes of MTVT cases with local recurrence**

| Nr | **Autor (Year)** | **Age, Side** | **Size (mm)** | **Location** | **Primary Treatment** | **Histology** | **Time to recurrence (months)** | **Laterality** | **Adjuvant Treatment** | **Surgical Salvage Treatment** | **Additional Treatment after Recurrence** | **Second Recurrence** | **Treatment of second recurrence** | **(Mean) follow up time (months)** | **Outcome during follow up** |
| --- | --- | --- | --- | --- | --- | --- | --- | --- | --- | --- | --- | --- | --- | --- | --- |
| 1 | Abello, A., et al. (2018) | 80, Right | 31 | TV + TA and testis | Orchiectomy | BT | 26 | Contralateral | AR | Orchiectomy | - | - | - | 26 | N/a |
| 2 | Amin, R. (1995) | 59, Right | N/a | TV only | Orchiectomy | BT | 39 | Ipsilateral | AR | Resection | Radio | Yes | Resection | 192 | Death |
| 3 | Bass, L. and T. W. Hegeman (2011) | 64, Left | N/a | TV only | Orchiectomy, RPLND | UK | 20 | Ipsilateral | HS, ILND | Resection | - | Yes | Radio | N/a | AWD |
| 4 | Bertolotto, M., et al. (2016) | 65, Left | N/a | TV only | Orchiectomy | ET | 48 | N/a | - | - | - | - |  | 132 | DOD |
| 5 | Bisceglia, M., et al. (2010) | 74, Right | N/a | TV + TA and testis, epididymis, spermatic cord | Orchiectomy | ET | 24 | Ipsilateral | - | HS | - | Yes | Resection | 102 | AWD |
| 6 | Carp, N. Z., et al. (1990)  *also third recurrence | 54, Left | N/a | TV + spermatic cord, scrotum | Orchiectomy | ET | 11 | Ipsilateral | - | - | Chemo (CR) | Yes | Chemo, HS, resection (left lower abdominal wall) | 68 | DOD |
| 7 | Chien, A. J., et al. (2000) | 16, Right | 20 | TV + TA and testis | TSS | BCM | 12 | N/a | - | Orchiectomy | - | - | - | 24 | N/a |
| 8 | Eden, C. G., et al. (1995) | 76, Right | N/a | TV only | Orchiectomy | UK | 8 | Ipsilateral (1) and Contralateral (2) | - | Resection (1) | - | Yes | Resection (2) | 27 | NED |
| 9 | Gonzalez, S. M., et al. (2017) | 83, Right | N/a | TV + TA and testis | Orchiectomy | ET | 24 | Ipsilateral | - | - | - | - | - | 48 | Death |
| 10 | Gupta, N. P., et al. (1999) | 69, Right | N/a | TV only | Orchiectomy | BT | 1 | Ipsilateral | AC (PD) | - | Radio (PD) | - | - | 18 | DOD |
| 11 | Gurdal, M. and A. Erol (2001) | 67, Right | N/a | TV + TA and testis, epididymis, spermatic cord | HSwO | ET | 24 | N/a | - | Resection | - | - | - | 30 | NED |
| 12 | Hai, B., et al. (2012) | 67, Left | N/a | TV only | Orchiectomy, ILND | ET | N/a | N/a | AC | - | - | - | - | 24 | AWD |
| 13 | Hai, B., et al. (2012) | 57, Right | N/a | TV + Epididymis, spermatic cord | TSS | ET | N/a | N/a | AC, AR | - | - | - | - | 24 | AWD |
| 14 | Hai, B., et al. (2012) | 78, Left | N/a | TV only | Orchiectomy | ET | N/a | N/a | - | - | - | - | - | 24 | AWD |
| 15 | Iczkowski, K. A., et al. (2002) | 71, Left | N/a | TV only | HSwO | ET | 19 | Ipsilateral | - | Resection | - | - | - | 26 | Death |
| 16 | Jankovichova, T., et al. (2015) | 67, Left | N/a | TV + TA and testis | Orchiectomy | ET | 14 | Ipsilateral | - | Resection | - | - | - | 44 | NED |
| 17 | Jones, M. A., et al. (1995) | 50, N/a | N/a | TV only | - | ET | 24 | N/a | - | - | - | - | - | N/a | N/a |
| 18 | Jones, M. A., et al. (1995) | 65, N/a | N/a | TV + Infiltration to spermatic cord | Orchiectomy | BT | 180 | N/a | - | - | - | - | - | 180 | N/a |
| 19 | Kanazawa, S., et al. (1999) | 68, Bilateral | 120 | TV + spermatic cord | TSS (Right) | ET | 36 | Contralateral | - | Orchiectomy | - | - | - | 156 | NED |
| 20 | Khor, H. Y., et al. (2018) | 34, Right | N/a | TV + spermatic cord | TSS | UK | 24 | Ipsilateral | - | Resection | - | - | - | 24 | N/a |
| 21 | Lee, M., et al. (1998) | 45, Right | N/a | TV + scrotum | Orchiectomy | UK | 4 | Contralateral | Sec. ILND | - | Chemo (PD) | - | - | N/a | DOD |
| 22 | Leite, K. R. M., et al. (2002) | 74, Left | 55 | TV + TA and testis, epididymis, spermatic cord | Orchiectomy | ET | 2 | N/a | - | HS | - | - | - | 2 | N/a |
| 23 | Liguori, G., et al. (2007) | 68, Left | 70 | TV + epididymis | TSS | UK | 24 | Ipsilateral | - | Orchiectomy | - | Yes | Resection | 84 | NED |
| 24 | Lihuan, D., et al. (2018) | 58, Left | N/a | TV + epididymis | Orchiectomy | ET | 10 | Ipsilateral | - | Resection | - | - | - | 12 | NED |
| 25 | Maheshwari, P., et al. (2017) | 20, Left | 18 | TV only | Orchiectomy | UK | 1 | N/a | - | - | Chemo (PR) | - | - | 16 | DOD |
| 26 | Melhouf, M. M., et al. (1998) | 65, Right | 50 | TV only | Orchiectomy | BT | 3 | N/a | - | Resection | - | Yes | Resection | 9 | DOD |
| 27 | Menut, P., et al. (1996) | 71, Right | N/a | TV + spermatic cord, scrotum | HSwO | UK | 55 | Contralateral | AR | - | Chemo (PD) | - | - | N/a | N/a |
| 28 | Mrinakova, B., et al. (2016) | 67, Left | N/a | TV + TA and testis | Orchiectomy | ET | 24 | Ipsilateral | - | Resection | - | - | - | 62 | NED |
| 29 | Pannier, D., et al. (2011) | 65, Left | N/a | TV only | Orchiectomy | ET | 36 | N/a | - | Resection | - | Yes | Resection | 71 | DOD |
| 30 | Park, Y. J., et al. (2011) | 65, Left | 60 | TV + spermatic cord | Orchiectomy, ILND | BT | 3 | Contralateral | - | - | Chemo (PD) | - | - | 6 | DOD |
| 31 | Poggi, A., et al. (2000) | 47, Right | N/a | TV only | TSS | ET | 1 | N/a | - | - | Chemo (PD) | - | - | 8 | DOD |
| 32 | Priester, P., et al. (2012) | 71, Right | N/a | TV only | TSAO | MUMP | 17 | N/a | - | Resection | - | - | - | 24 | DOD |
| 33 | Sebbag, G., et al. (2001) | 19, Links | N/a | TV + spermatic cord | Orchiectomy | ET | 11 | Ipsilateral | - | HS+ILND | Chemo (PD) | - | - | 24 | DOD |
| 34 | Shimada, S., et al. (2004) | 64, Right | 50 | TV + scrotum | Orchiectomy | BT | 1 | Ipsilateral | - | Resection | Chemo (PR) | - | - | 18 | AWD |
| 35 | Umekawa, T. and T. Kurita (1995) | 67, Right | N/a | TV + spermatic cord | Orchiectomy | ET | 4 | Ipsilateral | RPLND | - | Radio (CR) | Yes | Chemo (PD) | 8 | DOD |

*N/a: No information available, TV: Tunica vaginalis, TA: Tunica albuginea, TSS: Testis sparing surgery, HSwO: Hemiscrotectomy with Orchiectomy, HS: Hemiscotectomy, TSAO: Transscrotal approach orchiectomy, Radio: Radiotherapy, AR: Adjuvant Radiotherapy, Chemo: Chemotherapy, AC: Adjuvant Chemotherapy, RPLND: Retroperitoneal lymph node dissection, ILND: inguinal lymph node dissection, ET: Epitheloid type, BT: Biphasic type, UK: Malignant Mesothelioma of unknown type, BCM: Benign cystic mesothelioma, MUMP: Mesothelioma of uncertain malignant potential, AWD: Alive with disease, DOD: Dead of disease, NED: No evidence of disease, CR: Complete Remission, PR: Partial Remission, SD: Stable Disease, PD: Progressive Disease*
