# Supplementary material for: Clinicopathological characteristics and outcomes in men with mesothelioma of the tunica vaginalis testis: analysis of published case-series data
Source: J Cancer Res Clin Oncol. 2021 Feb 9;147(9):2671–9. doi: 10.1007/s00432-021-03533-6 (PMC8310841; doi:10.1007/s00432-021-03533-6)
Supplement: Supplementary file 2 — Supplementary file2 (DOCX 46 KB) [file 432_2021_3533_MOESM2_ESM.docx]

**Supplementary table 2: Features and treatment outcomes of metastatic MTVT cases**

| **Nr** | **Autor** | **Year** | **Age** | **Side** | **Histologic Type** | **Size (mm)** | | **Location** | **Primary treatment** | **Initial metastatic site (months)** | **Additional site (months)** | **First-line treatment** | **Response to first-line treatment** | **Second-line treatment** | **Response to second-line treatment** | **Local recurrence** | **Risk Factors** | **Follow up (months)** |
| --- | --- | --- | --- | --- | --- | --- | --- | --- | --- | --- | --- | --- | --- | --- | --- | --- | --- | --- |
| 1 | Maheshwari, P., et al. | 2017 | 20 | Left | MM of Unknown Type | | 18 | TV only | Orchiectomy | RPLN (N/a) | - | Chemo (AM), RT | - | - | - | Yes | - | DOD (16) |
| 2 | Gonzalez, S. M., et al. | 2017 | 83 | Right | Epithelioid | | N/a | TV + TA and Testis | Orchiectomy | RPLN/Skin (24) | - | N/a | - | - | - | Yes | Age+, ALI | Death (48) |
| 3 | An, J. Y., et al. | 2017 | 43 | Right | Epithelioid | | N/a | TV only | HSwO | Peritoneal (12) | - | N/a | - | - | - | - | Age+ | DOD (60) |
| 4 | Serio, G., et al. | 2016 | 77 | Left | Epithelioid | | 100 | TV + Epididymis  + TA and Testis  + Scrotum | HSwO | RPLN/Abdominal LN (26) | - | Chemo (PEM & CIS) | PR | - | - | - | Age+, Size+, ALI | DOD (44) |
| 5 | Hispan, P., et al. | 2016 | 93 | Left | Epithelioid | | 50 | TV + Scrotum | Palliative | RPLN/ILN/Lungs/Skin (0) | - | Not considered | - | - | - | - | Age+, Size+, ALI | LTF |
| 6 | Bertolotto, M., et al. | 2016 | 75 | Right | MM of Unknown Type | | N/a | TV only | N/A | RPLN (N/a) | - | N/a | - | - | - | - | Age+ | N/a |
| 7 | Bertolotto, M., et al. | 2016 | 60 | Left | Epithelioid | | N/a | TV only | Orchiectomy + RPLND (pos.) | RPLN (0) | - | N/a | - | - | - | - | Age+ | NED (66) |
| 8 | Bertolotto, M., et al. | 2016 | 65 | Left | Epithelioid | | N/a | TV only | Orchiectomy | SCLN (84) | RPLN/Lungs (108) | N/a | - | - | - | Yes | Age+ | DOD (132) |
| 9 | Bertolotto, M., et al. | 2016 | 70 | Right | Epithelioid | | N/a | TV only | Orchiectomy | RPLN/Lungs/ Liver (12) | - | N/a | - | - | - | - | Age+ | Death (24) |
| 10 | Bertolotto, M., et al. | 2016 | 63 | Bilat. | MM of Unknown Type | | N/a | TV only | N/A | RPLN/Peritoneal  (N/a) | - | N/a | - | - | - | - | Age+ | N/a |
| 11 | Segura-Gonzalez, M., et al. | 2015 | 58 | Left | Epithelioid | | N/a | TV + Epididymis | Orchiectomy | Rectal/Vesical (N/a) | - | Chemo (GEM & CAR), RT | SD | - | - | - | Age+ | AWD (6) |
| 12 | Doris, M., et al. | 2015 | 73 | Right | Epithelioid | | N/a | TV only | Orchiectomy | MLD (36) | - | Chemo (PEM & CIS) | CR | PEM | - | - | Age+ | NED (42) |
| 13 | Hebbar, A., et al. | 2014 | 54 | Left | Biphasic | | 120 | TV only | Orchiectomy | ILN/ILLN (0) | - | N/a |  | - | - | - | Age+, Size+ | LTF |
| 14 | da Fonseca, L. G., et al. | 2014 | 62 | Left | Biphasic | | N/a | TV + Epididymis  + TA and Testis  + Spermatic Cord | Orchiectomy | RPLN/ILN/ILLN/MLD/Lungs (3) | - | Chemo (PEM & CIS) | PD | - | - | - | Age+, ALI | DOD (5) |
| 15 | Rajan, V., et al. | 2013 | 18 | Left | MM of Unknown Type | | N/a | TV only | Orchiectomy | Bone/Brain (10) | - | Chemo (AM & PAC) | - | RT | PD | - | - | DOD (14) |
| 16 | Priester, P., et al. | 2012 | 71 | Right | MM of uncertain malignant potential | | N/a | TV only | Transscrotal Orchiectomy | ILN/ Pelvic cavity (17) | Skin (23) | Resection, Chemo (PEM & CIS), RT | SD | Chemo (PEM & CAR) | PD | Yes | Age+ | DOD (24) |
| 17 | Hai, B., et al. | 2012 | 57 | Right | Epithelioid | | N/a | TV + Epididymis  + Spermatic Cord | TSS | Lungs (N/a) | - | N/a | - | - | - | Yes | Age+ | AWD (24) |
| 18 | Hai, B., et al. | 2012 | 46 | Left | Epithelioid | | N/a | TV only | Orchiectomy | RPLN (N/a) | - | N/a | - | - | - | - | Age+ | DOD (24) |
| 19 | Hai, B., et al. | 2012 | 78 | Left | Epithelioid | | N/a | TV only | Orchiectomy | ILN (N/a) | - | N/a | - | - | - | Yes | Age+ | AWD (24) |
| 20 | Hai, B., et al. | 2012 | 76 | Right | Epithelioid | | N/a | TV only | Orchiectomy | RPLN/Lungs (N/a) | - | N/a | - | - | - | - | Age+ | DOD (24) |
| 21 | Borghesi, M., et al. | 2012 | 67 | Right | Biphasic | | N/a | TV + TA and Testis | Orchiectomy | Lungs/Bone/Liver/Pancreas (0) | - | N/a | - | - | - | - | Age+ | N/a |
| 22 | Park, Y. J., et al. | 2011 | 65 | Left | Biphasic | | 60 | TV + Spermatic Cord | Orchiectomy + ILND (pos.) | ILN, Peritoneal (3) | - | Chemo (CIS) | PD | - | - | Yes | Age+, Size+ | DOD (6) |
| 23 | Pannier, D., et al. | 2011 | 65 | Left | Epithelioid | | N/a | TV only | Orchiectomy | Pleura/Lungs (60) | - | Chemo (PEM & CIS) | PD | - | - | Yes | Age+ | DOD (71) |
| 24 | Bass, L. and T. W. Hegeman | 2011 | 64 | Left | MM of Unknown Type | | N/a | TV only | Orchiectomy + RPLND (neg.) + INLD (neg.) | Peritoneal/Lungs (44) | - | Chemo | SD | RT | - | Yes | Age+ | AWD (N/a) |
| 25 | Bisceglia, M., et al. | 2010 | 74 | Right | Epithelioid | | N/a | TV + Epididymis  + TA and Testis  + Spermatic Cord | Orchiectomy | RPLN (24) | ILN (72), Pelvic cavity/MLN/SCLN/Lungs (96), Skin (108) | RPLND, ILND, Chemo, RT | - | Chemo (PEM & CAR), RT | - | Yes | Age+ | AWD (108) |
| 26 | Aggarwal, P., et al. | 2010 | 75 | Left | MM of Unknown Type | | N/a | TV only | Orchiectomy | RPLN (30) | Abdominal LN/Lungs/Skin/Liver (54) | RPLND, RT | PD | - | - | - | Age+ | DOD (76) |
| 27 | Bacchetta, J., et al. | 2009 | 63 | Right | Epithelioid | | N/a | TV only | TSS | RPLN (17) | Bone (28), Peritoneal (32) | RPLND, Chemo (PEM & CIS), RT | PD | - | - | - | Age+, ALI | DOD (33) |
| 28 | Muller, M., et al. | 2008 | 74 | Left | Epithelioid | | N/a | TV + Epididymis  + TA and Testis  + Spermatic Cord + Scrotum | Orchiectomy + ILND | Pleura (0) | - | Chemo (GEM & CIS) | - | - | - | - | Age+, ALI | DOD (7) |
| 29 | Mathur, S. R., et al. | 2008 | 65 | Left | Epithelioid | | 110 | TV + TA and Testis + Spermatic Cord | Orchiectomy | RPLN (0) | - | N/a | - | - | - | - | Age+, Size+ | LTF |
| 30 | Ikegami, Y., et al. | 2008 | 67 | Right | MM of Unknown Type | | N/a | TV + Scrotum | HSwO | Liver (24) | - | N/a | - | - | - | - | Age+ | DOD (26) |
| 31 | Boyum, J. and N. F. Wasserman | 2008 | 60 | Left | Biphasic | | 40 | TV only | HSwO | RPLN/MLN/SCLN/Lungs (N/a) | - | RPLND, Chemo | - | Chemo | - | - | Age+ | AWD |
| 32 | Barui, G. N., et al. | 2008 | 42 | Right | Biphasic | | 70 | TV only | Orchiectomy | ILN (N/a) | - | N/a | - | - | - | - | Age+, Size+, ALI | N/a |
| 33 | van Apeldoorn, M. J., et al. | 2006 | 83 | Right | Biphasic | | N/a | TV only | Orchiectomy | RPLN (6) | - | N/a | - | - | - | - | Age+ | DOD (6) |
| 34 | Schure, P. J., et al. | 2006 | 36 | Left | MM of Unknown Type | | N/a | TV + Spermatic Cord | HSwO | Peritoneal/ Pleura (N/a) | - | RPLND, Chemo | PD | - | - | - | Age+, ALI | DOD (N/a) |
| 35 | Schure, P. J., et al. | 2006 | 26 | Left | MM of Unknown Type | | N/a | TV + Spermatic Cord | Laparascopy & TSS | Peritoneal (0) | Pelvic cavity/Abdominal LN (3) | Peritonec-tomy,intrape-ritoneal hyperthermic perfusion | - | - | - | - |  | NED (18) |
| 36 | Hatzinger, M., et al. | 2006 | 76 | Right | Epithelioid | | N/a | TV + Epididymis  + TA and Testis | Orchiectomy | Peritoneal (18) | - | N/a | - | - | - | - | Age+ | DOD (18) |
| 37 | Shimada, S., et al. | 2004 | 64 | Right | Biphasic | | 50 | TV + Infiltration of external scrotal layer | Orchiectomy | Peritoneal (12) | RPLN (16) | Resection, Chemo | | Chemo | - | Yes | Age+, Size+ | AWD (18) |
| 38 | Garcia de Jalon, A., et al. | 2003 | 78 | Right | Biphasic | | N/a | TV + Epididymis  + TA and Testis | Orchiectomy | RPLN (0) | - | RT | - | - | - | - | Age+ | AWD (3) |
| 39 | Black, P. C., et al. | 2003 | 67 | Right | MM of Unknown Type | | N/a | TV only | HSwO | ILN/Pelvic cavity (3) | Skin/Lungs (N/a) | ILND, RT | - | Chemo (GEM & CIS) | PR | - | Age+ | DOD (48) |
| 40 | Iczkowski, K. A., et al. | 2002 | 71 | Left | Epithelioid | | N/a | TV only | HSwO | Liver (19) | - | Treatment refused | - | - | - | Yes | Age+ | Death (26) |
| 41 | Abe, K., et al. | 2002 | 81 | Left | Epithelioid | | N/a | TV only | Orchiectomy | RPLN/Bone (13) | - | Treatment refused | - | - | - | - | Age+ | DOD (13) |
| 42 | Sebbag, G., et al. | 2001 | 34 | Left | Epithelioid | | N/a | TV + Spermatic Cord | Laparascopy & HSwO | Pelvic cavity/Peritoneal/Colon (0) | - | Resection with extensive peritonectomy, perioperative chemo (CIS, DOX) | - | - | - | - | - | NED (60) |
| 43 | Sebbag, G., et al. | 2001 | 19 | Left | Epithelioid | | N/a | TV + Spermatic Cord | Orchiectomy | ILN (11) | Lungs (24) | ILND, Chemo (DOX) | PD | - | - | Yes | ALI | DOD (24) |
| 44 | Schneider, J. and H. J. Woitowitz | 2001 | 53 | Left | Epithelioid | | N/a | TV only | Orchiectomy + RPLND | RPLN/MLN/Lungs (12) | - | N/a | - | - | - | - | Age+ | N/a |
| 45 | Uria Gonzalez-Tova, J., et al. | 2000 | 62 | Left | Epithelioid | | N/a | TV + Epididymis  + TA and Testis  + Infiltration of external scrotal layer | HSwO | Lungs/Skin/Liver (N/a) | - | Chemo, RT | PD | - | - | - | Age+ | N/a |
| 46 | Poggi, A., et al. | 2000 | 47 | Right | Epithelioid | | N/a | TV only | TSS | Peritoneal/Pleura/Liver (1) | - | Chemo (CIS, AM, CYCLP) | PD | Chemo (AM, IFO, MESNA) | PD | Yes | Age+ | DOD (8) |
| 47 | Leiber, C., et al. | 2000 | 46 | Right | Biphasic | | 90 | TV only | Orchiectomy | Peritoneal (N/a) | - | Resection, Chemo (INT) |  | - | - | - | Age+, Size+ | DOD (8) |
| 48 | Ferri, E., et al. | 2000 | 64 | N/a | Epithelioid | | N/a | TV + Epididymis  + TA and Testis | Orchiectomy | Pleura (0) | - | Chemo (CYCLP) | PD | - | - | - | Age+ | DOD (1) |
| 49 | Attanoos, R. L. and A. R. Gibbs | 2000 | 77 | Left | Epithelioid | | 5 | TV + TA and Testis | Orchiectomy | Pleura (28) | - | N/a | - | - | - | - | Age+ | DOD (50) |
| 50 | Gupta, N. P., et al. | 1999 | 51 | Left | Biphasic | | 100 | TV + Spermatic Cord | Orchiectomy | ILN/ILLN (0) | Multiple – Locations unknown (Widespreading) (5) | Chemo (EPI, CIS, IFO) | - | - | - | - | Age+, Size+ | DOD (5) |
| 51 | Melhouf, M. M., et al. | 1998 | 65 | Right | Biphasic | | 50 | TV only | Orchiectomy | Brain (8) | - | RT | PD | - | - | Yes | Age+, Size+ | DOD (9) |
| 52 | Lee, M., et al. | 1998 | 45 | Right | MM of Unknown Type | | N/a | TV + Infiltration of external scrotal layer | Orchiectomy | ILN (4) | - | RT | PD | - | - | Yes | Age+, ALI | DOD (4) |
| 53 | Gupta, S. C., et al. | 1998 | 36 | Right | Biphasic | | 4 | TV only | Orchiectomy | RPLN/ILN (8) | SCLN (9), CLN/Lungs (10) | N/a | - | - | - | - | ALI | Death (11) |
| 54 | Agapitos, E., et al. | 1997 | 60 | Left | Biphasic | | N/a | TV only | Orchiectomy | Liver (15) | - | Chemo (CIS) | PD | - | - | - | Age+ | Death (20) |
| 55 | Menut, P., et al. | 1996 | 71 | Bilat | MM of Unknown Type | | N/a | TV + Infiltration of external scrotal layer | HSwO | RPLN/Pelvic cavity (86) | - | RT | PD | - | - | Yes | Age+, ALI | AWD |
| 56 | Mathew, B. S., et al. | 1996 | 70 | Left | MM of Unknown Type | | N/a | TV only | Orchiectomy | Bone (0) | - | RT | PD | - | - | - | Age+ | DOD (3) |
| 57 | Mathew, B. S., et al. | 1996 | 58 | Right | MM of Unknown Type | | N/a | TV only | Orchiectomy + ILND (pos.) | ILN/ILLN/Bone (0) | - | Chemo (EPI), RT | PD | - | - | - | Age+ | DOD (2) |
| 58 | Ascoli, V., et al. | 1996 | 55 | Right | Epithelioid | | N/a | TV only | N/A | Peritoneal/ Pleura (0) | - | - |  | - | - | - | Age+ | DOD (6) |
| 59 | Umekawa, T. and T. Kurita | 1995 | 67 | Right | Epithelioid | | 26 | TV + Spermatic Cord | Orchiectomy | RPLN (4) | Lungs (6) | RT | PD | Chemo (CIS & DOX) | SD | Yes | Age+ | DOD (8) |
| 60 | Lopez, J. I., et al. | 1995 | 47 | Left | Epithelioid | | 150 | TV + TA and Testis + Spermatic Cord + Infiltration of external scrotal layer | HSwO + RPLND | RPLN (0) | - | Chemo (AM, CYCLP), RT | CR | - | - | - | Age+, Size+, ALI | NED (36) |
| 61 | Loizaga, A., et al. | 1995 | 50 | Left | MM of Unknown Type | | N/a | TV + TA and Testis | HSwO | RPLN/ILN (0) | Pleura/Lungs (6), Peritoneal (18) | RPLND, ILND, Chemo (AM, CYCLP), RT | PR | Chemo (CIS, IFO, MESNA, EPI) | PD | - | Age+ | DOD (18) |
| 62 | Joseph, A. K., et al. | 1995 | 26 | Right | Epithelioid | | N/a | TV only | Orchiectomy | ILN (0) | Skin (144) | ILND | - | - | - | - | - | AWD (12) |
| 63 | Amin, R. | 1995 | 59 | Right | Biphasic | | N/a | TV only | Orchiectomy | Lungs (78) | MLN/Skin (110) | Resection, RT | CR | Chemo, RT | PR | Yes | Age+ | Death (192) |
| 64 | Reynard, J. M., et al. | 1994 | 76 | Right | Biphasic | | 165 | TV + TA and Testis | HSwO | Lungs (0) | - | N/a | - | - | - | - | Age+, Size+ | N/a |
| 65 | Kuwabara, H., et al. | 1991 | 60 | Right | Biphasic | | N/a | TV only | Orchiectomy | RPLN/MLN/Peritoneal/Pleura/Lungs/Kidney/Pancreas/Spleen/Bladder (N/a) | - | N/a | - | - | - | - | Age+, ALI | DOD (5) |
| 66 | Smith, J. J., 3rd, et al. | 1990 | 57 | Left | Epithelioid | | N/a | TV only | HSwO + RPLND (pos.) + ILND (pos.) | SCLN (40) | - | Chemo | - | - | - | - | Age+, ALI | AWD (N/a) |
| 67 | Rodriguez Andres, J. A., et al. | 1990 | 73 | Right | Biphasic | | N/a | TV + Epididymis | Orchiectomy | Pleura (0) | - | N/a | - | - | - | - | Age+ | DOD (2) |
| 68 | Carp, N. Z., et al. | 1990 | 54 | Left | Epithelioid | | N/a | TV + Spermatic Cord + Infiltration of external scrotal layer | Orchiectomy | Pelvic cavity/Abdominal LN (11) | ILN (45) | RT | CR | Chemo (CIS) | - | Yes | Age+ | DOD (68) |

| *N/A: No information available, TV: Tunica vaginalis, HSwO: Hemiscrotectomy with Orchiectomy, TSS: Testis Sparing Surgery, LN: Lymph Nodes, RPLN: Retroperitoneal Lymph Nodes, ILN: Inguinal Lymph Nodes, ILLN: Iliacal Lymph Nodes, SCLN: Supraclavicular Lymph Nodes, MLN: Mediastinal Lymph Nodes, RPLND: Retroperitoneal Lymph Node Dissection, ILND: Inguinal Lymph Node Dissection, RT: Radiotherapy,*  *Chemo: Chemotherapy; AM: Adriamycin, PEM: Pemetrexed, CIS: Cisplatin, GEM: Gemcitabin, CAR: Carboplatin, PAC: Paclitaxel, CYCLP: Cyclophosphamid, INT: Interferon, EPI: Epirubicin, DOX: Doxorubicin, IFO: Ifosphamid,*  *CR: Complete Remission, PR: Partial Remission, SD: Stable Disease, PD: Progressive Disease, ALI: Angiolymphatic Invasion, NED: No Evidence of Disease, DOD: Died of Disease, AWD: Alive with Disease, LTF: Lost to Follow up* |
| --- |
