# Supplementary material for: Clinicopathological characteristics and outcomes in men with mesothelioma of the tunica vaginalis testis: analysis of published case-series data
Source: J Cancer Res Clin Oncol. 2021 Feb 9;147(9):2671–9. doi: 10.1007/s00432-021-03533-6 (PMC8310841; doi:10.1007/s00432-021-03533-6)

Suchresultate zur Studie `Treatment Outcomes in patients with Localised and Metastatic Testicular and Paratesticular Sarcoma and Mesothelioma`

Suchprotokolle:

- ClinicalTrials 14012019.pdf
- Cochrane 14012019.pdf
- Embase 14012019.pdf
- Medline 14012019.pdf
- Scopus 14012019.pdf
- Web of Science 14012019.pdf

	**Deduplication**	
	Before	after
Medline	1615	1608
Embase	2810	1426
Cochrane	29	13
Scopus	2307	379
Web of Science	1373	307
ClinicalTrials	54	53
Pool	8188	**3787**

Reference files: testicular sarcoma mesothelioma.enlx

Before:

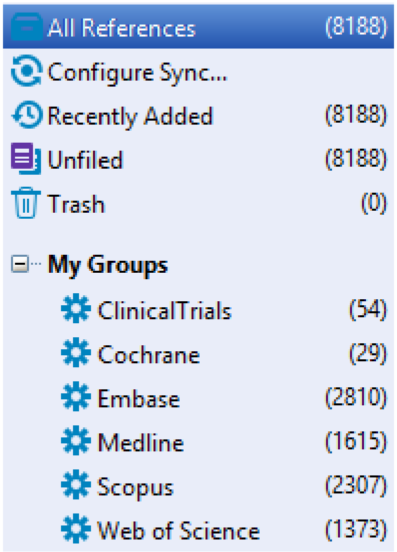

after:

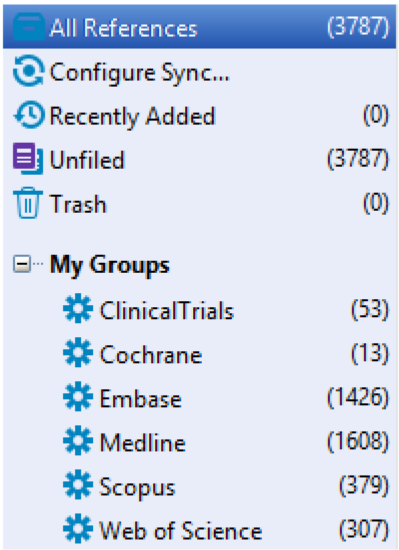

Supplement: Supplementary file 3 — Supplementary file3 (PDF 60 KB) [file 432_2021_3533_MOESM3_ESM.pdf]
